# Survey of abuses against injecting drug users in Indonesia

**DOI:** 10.1186/1477-7517-6-28

**Published:** 2009-10-24

**Authors:** Sara LM Davis, Agus Triwahyuono, Risa Alexander

**Affiliations:** 1 Asia Catalyst, PO Box 20839, New York, NY 10009, USA; 2 JANGKAR, Jalan Haji Nawi No. 12, RT 01/02, Gandaria, Jakarta 12430, Indonesia

## Abstract

In Indonesia, an ongoing government "war on drugs" has resulted in numerous arrests and anecdotal reports of abuse in detention, but to date there has been little documentation or analysis of this issue. JANGKAR (also known in English as the Indonesian Harm Reduction Network), a nongovernmental organization (NGO) based in Jakarta, surveyed 1106 injecting drug users in 13 cities about their experiences of police abuse. Of those interviewed, 667 or 60% reported physical abuse by police. These findings indicate the importance of continuing efforts to promote police reform and harm reduction in Indonesia.

## Background

The government estimates that there are 200,000 injecting drug users in Indonesia, but grassroots NGOs estimate the number to be higher[1]. There are currently an estimated 270,000 people living with HIV in Indonesia[2]. Low-grade heroin, or *putau*, took hold in Indonesia in the 1990s[3]. The rapid increase in injection drug use since that time has led to an increase in HIV prevalence in Indonesia[4]. According to the National AIDS Commission, HIV prevalence is 46-48% of injecting drug users[5]. Over half of those living with HIV are people who inject drugs[6].

Indonesia's response to drug use, like that of many other countries, began with a punitive approach, with the launch in 1997 of a national war on drugs, which included the use of the death penalty in drug-related cases[7]. In 2000, Indonesia was a signatory to the ASEAN pledge to pursue a "Drug-Free ASEAN" by 2015[8].

Indonesia's "war on drugs" has included sweeping arrests and lengthy prison sentences for both traffickers and individuals found in possession of narcotics. Those found guilty of trafficking face more than nine years in prison or, in certain circumstances, the death sentence[9]. Those found in possession of even a small amount of narcotics may serve up to nine years in prison, including pre-trial detention periods that can last months. Indonesian laws do not provide guidelines for sentencing based on the amount of narcotics in possession, so judges exercise wide discretion in drug cases, often issuing draconian sentences. Those found guilty of trafficking face over nine years in prison or the death sentence. Those found in possession of even a small amount of narcotics may serve up to nine years in prison, after periods of pre-trial detention that can spread into months. Indonesian laws do not provide guidelines for sentencing based on the amount of narcotics in possession, so judges have had broad leeway to hand down heavy sentences[10]. Despite these harsh measures, according to the BNN, the number of drug-related criminal cases increased from 17,355 in 2006 to 22,630 cases in 2008[11].

At the same time, incipient harm reduction efforts have begun to scale up, while sometimes operating in tension with law enforcement. Significant international support has helped to facilitate the growth and development of dozens of NGOs at the community level providing harm reduction services, many of them founded and staffed by former drug users. In 2007, sixteen of these groups were providing clean needles to approximately 4,500 drug users, and both numbers are probably higher today. Over 100 community health centers, or *puskesmas*, also offer needle and syringe exchange services[12].

The first prison methadone maintenance program was established in Kerobokan Prison in Bali in 2006; today it is offered in four prisons, and VCT is provided in prisons in thirteen provinces[13,14]. Currently, no prisons offer needle exchanges[15]. While international donors have provided training in HIV and drug use issues to prison staff, harm reduction programs in prison are hampered by the severe lack of funding for health services in general[16].

As in many other countries, efforts to promote harm reduction and to train police in harm reduction principles run into roadblocks when police are simultaneously ordered to implement draconian "drug war" policies and laws.

## Methods

Jaringan Aksi Nasional Penguran Dampak Buruk Narkoba Suntik, or JANGKAR, is a national network of local nonprofits that work with injecting drug users. Research into police torture of injecting drug users was conducted between September 26, 2007 and February 28, 2008.

Field researchers utilized convenience sampling and street-based outreach to recruit participants for this study. Eligibility criteria included self-identification as a current injecting drug user and provision of informed consent. At study entry, each participant completed an interviewer-administered questionnaire that included biodata such as age, gender, occupation, educational level, and marital status. Researchers explained the purpose of the research report to interviewees and obtained their consent. Interviews were conducted anonymously and each questionnaire was assigned a number; research data were kept locked in a secure location.

Subjects were asked if they had experienced either police abuse or discrimination in access to health services. Those who replied in the affirmative were asked to give testimony about the incident or incidents. Some respondents declined to discuss the incidents in more depth because they were reluctant to recall traumatic experiences.

A second interviewer-administered questionnaire asked for the date, time, perpetrator, place of the abuse, and details of the abuse. This questionnaire also asked victims to describe the physical, psychological and social effects of the abuse. Interviewers used audio recordings to back up their written interviews.

Researchers underwent training by three experts in research, including Willy Aditya, Research and Development Coordinator for the Voice of Human Rights; Binoto Nadapdap, a lecturer from the University of Indonesia; and a third research specialist who requested anonymity for this article.

## Findings

A total of 1106 participants completed the baseline survey and were eligible for this study. 985 (89%) were male. 987 (89%) had a senior high school education or above. 668 (60%) were single, never married. 409 (37%) respondents were unemployed, and 487 (44%) described themselves as self-employed.

Of the entire sample, 667 or 60% reported physical abuse by police. These incidents included beating of the feet, hands, chest, and head by officers using their hands, fists, and boots. In addition, several subjects said that police had beaten them with pistol butts, folded chairs, or blackjacks. One reported being beaten with a wrench and the flat side of a metal saw. Other abuses reported included being burned with cigarettes or given an electrical shock. 66 (6%) respondents reported sexual harassment or abuse, including inappropriate touching of women during street searches by male police officers.

Such physical abuse is clearly prohibited by both international and domestic laws. Indonesia is party to the major United Nations human rights treaties that prohibit torture and other forms of cruel, inhuman or degrading treatment or punishment. These include the *International Covenant on Civil and Political Rights (ICCPR)*, and the *Convention against Torture and Other Cruel, Inhuman or Degrading Treatment or Punishment (CAT)*[17]. Indonesia's Constitution also stipulates that "each person has the right to be free from torture or inhuman and degrading treatment"[18].

*Confessions and corruption *The JANGKAR questionnaires did not segment out motives or causes of abuse, which in some cases might be multiple, and in other cases unclear to the victims. However, based on a review of the individual reports, two motivations were important: the desire by police to obtain confessions and use these to ensure convictions, and the desire to extort bribes.

Confessions are acceptable evidence in courts in Indonesia, and the sense of urgency created by a war on drugs may be creating pressures on police to increase their numbers of convictions. One man interviewed on November 7, 2007 described his arrest in Jakarta:

Around 11 p.m. two policemen arrested me [at my friend's house]. They accused me of selling *putau *[low-grade heroin]. They searched me and my friend. They found nothing. After 30 minutes, three more policemen came. One of them was the Unit Head. I was taken to a car. In the car I was beaten up and my toenails were pulled out so that I would admit that I sold *putau*. It lasted four hours.

Another man interviewed in Semarang, Central Java, on November 1, 2007 said that he and his friends were beaten in an effort to compel them to sign a false confession:

My eyes were covered with a bandage and I was taken to the police station. All of us were beaten up...We were also treated rudely when they were preparing the official report. They beat us up with a chair, rattan stick, and an iron ruler. The official report they made was not in line with the real events. We were not accompanied by any lawyer during the process.

Another leading cause for police abuse appears to be the extortion of bribes. In 2007, Transparency International surveyed roughly one thousand Indonesian citizens and found that a majority said the police were the government agency most likely to take bribes, an assertion the police rejected[19]. Nonetheless, some of study respondents said that police beat them in order to extort "coordination fees," or bribes. In the words of a man Jangkar spoke with in Surabaya:

I was often arrested previously, but since there was no evidence I was released. My family often gave ransom to the police to release me. Each time I was caught by the police, I was tortured.

In Semarang, an injecting drug user reported to Jangkar that he was beaten, threatened with a gun to his head, and released when he paid 20 million rupiah. In a case from South Sumatra, police spotted a package of heroin sitting on the dashboard of a car and took the driver and his friend to the police station. In the words of the interviewee:

He asked for "coordination money" from us. Because we said that we had no money, he took our wallets. As he only got a little money from our wallets, he asked for [my friend's] watch. I threatened the officer that I would report him to my uncle, who has a higher rank, but [the officer] only punched me. After he took the *putaw *and the watch we were finally released.

NGOs working with drug users and organizations providing legal services to them report that corruption is widespread throughout the criminal justice system. Lawyers we spoke with at a human rights training for drug users in Jakarta said that drug users may be asked for bribes by prosecutors in exchange for lighter charges, and from judges in exchange for lighter sentences. Drug users who requested medical treatment for injuries sustained during interrogation said they were sometimes refused, as in the case of this man who spoke with Jangkar in Surabaya:

They tried to make me tell them where I bought the *putaw*. I refused to give any information. The police got impatient and hit me. One of the policemen folded a chair and used it to beat me all over my body. I broke my left hand. I gave the information eventually, because I could not stand it anymore.

On the way to the location [I had identified to the police], I begged them to take me to a hospital, but they laughed at me and said that I was fortunate that they had only broken my hand and did not shoot me in the kneecap.

Two respondents said that they were refused access to ARV treatment while in detention in the police station; they also said that police disclosed their HIV status to others.

In addition to physical abuse, respondents said that some police used threats to both drug users and their families, as well as public humiliation, to coerce confessions or extort bribes. Half of those interviewed reported some form of psychological abuse, including verbal abuse, being threatened with a gun, and other threats. In one case, an interviewee reported that he was forced to strip and submit to a search while standing on a public street.

Certain regions had a higher number of reported abuse incidents than others. In Jakarta, Denpasar, Medan and Kupang the percentage of respondents who experienced police abuse was especially high, over 85% (Table 1). However, the high number of respondents reporting abuse across the country collectively suggest a pattern that is broader than the problems of any single police station, city, or province.

**Table 1:** Geographic distribution of respondents

**Location**	**Number of respondents**	**Number who reported physical abuse**	**Percentage reporting physical abuse**
Medan	68	58	85%
Palembang	97	45	46%
Jakarta	117	111	95%
Bandung	108	51	47%
Semarang	83	54	65%
Yogyakarta	56	26	46%
Surabaya	61	32	53%
Samarinda	61	13	21%
Manado	100	33	33%
Makassar	100	48	48%
Denpasar	100	94	94%
Ambon	56	9	16%
Kupang	99	93	94%
**TOTAL**	**1106**	**667**	**60%**

Subsequent studies of police abuse of drug users have also found the problem to be widespread. In April 2009, the Indonesian Coalition for Drug Policy Reform published a report, based on interviews with injection drug users nine cities, indicating that almost all of those interviewed had suffered police abuse[20].

Other human rights reports have also found that the problem extends beyond drug users. In August 2008, the Jakarta Legal Institute (LBH), a leading Indonesian civil rights NGO, published a survey finding that a majority of those in police detention were subject to physical abuse[21]. And in November 2007, the UN Special Rapporteur on Torture conducted a mission to Indonesia, visiting police stations and prisons around the country and meeting with experts and nongovernmental organizations (NGOs). Torture is "routine practice" in Jakarta and other large cities in Java, he reported, and the conditions of detention in police stations amount to "degrading and inhuman treatment"[22]. The absence of transparency and monitoring systems to hold police accountable for torture results, he said, "in a system of quasi-total impunity."

## Discussion

The abuses documented in this study should be understood as both an outgrowth of Indonesia's conflicting policies toward drug users, and as part of the country's wider efforts to professionalize the police in the course of a national shift toward democratization. Police forces that, like Indonesia's, transition from military to civilian control are often plagued by reports of police abuse in the transitional periods[23].

However, the experience of drug users in detention also appears to be common among other detainees, and thus it is emblematic of deeper structural weaknesses within the police force and the legal system. Ending police abuse against drug users therefore involves not only promoting harm reduction policies, but also promoting deeper security sector reform, including reforming policies that incentivize abuse and establishing mechanisms that hold police accountable.

Beginning in the Dutch colonial era, the Indonesian police were a branch of the military. The military was in turn deeply enmeshed in the government bureaucracy, affiliated with political parties, and supervised extensive and lucrative business interests. The military, and the police as a part of the military, used intelligence services and force to advance their interests. Under the dictatorship period, the popular image of the military and the police in Indonesia was that they were corrupt, inefficient and brutal[24].

Since 1998, Indonesian police have participated in an extensive process of security sector reform[25]. Under this "New Paradigm" approach, the military has gradually removed itself from governance, and the police, in turn, have separated from the military. At the same time, the military and the police have been forced to give up a major source of revenue: their business interests in logging and other industries. The resulting lack of funding for police in Indonesia may be one cause of corruption.

The police have taken a number of sweeping steps towards reform: clarifying the respective functions of the military and the police; creating a national Police Law in 2002; creating new codes of conduct, uniforms and ranks; and extensively re-training police--overall an ambitious project, made even more challenging after the 2002 bombing of a Bali nightclub frequented by tourists created international pressure to combat terrorism[26].

Indonesia still needs to address some structural causes of police abuse. These include the over-reliance on confessions by Indonesia's judicial system, the lack of adequate government funding for police that could replace funds previously supplied through the military's business interests, and the lack of effective internal mechanisms to investigate complaints and hold police accountable for abuses. Indonesian police have been remarkably open to input in this process, for instance partnering with international donors and the International Office of Migration to hold human rights training for senior officers. However, some Indonesian experts have argued that security sector reform has been too "top-down" and focused on senior-level officers without addressing grass-roots police and their relationships with local communities[27].

*Ending abuse - root causes *Addressing the root causes of police abuse is a complex project, requiring multiple changes to police policies and to the larger legal framework.

Providing adequate funding to pay for police salaries can help to remove financial incentives for corruption. Reforming laws so that convictions may not be based on confessions alone can also remove a major motivation for torture of detainees; as can establishing a system for police evaluation and promotion that incorporates numbers of abuse complaints, and not simply numbers of arrests.

Experts who have examined successful efforts to eliminate police abuse in other countries also recommend establishing an independent and transparent civilian complaint mechanism[28]. Currently, there are two complaint mechanisms in Indonesia, but by most accounts, neither functions effectively. Recent high-level investigations of police misconduct appear to be undertaken selectively and without internal controls[29].

Finally, the detention and sentencing process should be reformed. One of the key factors often leading to abuse in detention is the length of the pre-sentencing detention period; the longer the detention, the more likely the abuse. U.N. Special Rapporteur on Torture Manfred Nowak recommends that in Indonesia,

As a matter of urgent priority, the period of police custody should be reduced to a time limit in line with international standards (maximum of 48 hours); after this period the detainees should be transferred to a pretrial facility under a different authority, where no further unsupervised contact with the interrogators or investigators should be permitted[30].

However, injection drug users in Indonesia can be legally detained for periods of up to eight or nine months before sentencing. As an Indonesian criminal lawyer (who requested his name be withheld from this article for security reasons) explained in discussion, if a suspect is facing a prison sentence of longer than nine years (i.e., for trafficking), she or he may be detained for up to 120 days, through a series of renewable detention periods. The prosecutor can then detain the suspect for up to an additional 110 days pending trial, and during the trial, the judge can detain the person for 150 days more. In a worst-case scenario, therefore, a suspect faces a potential detention period of eight to nine months before sentencing. In practice, lawyers say, most are detained for between two and four months.

Judges do have the option to sentence drug users to rehabilitation instead of prison. Until now, this has been an option judges rarely exercised, and there are only 45 drug rehabilitation facilities in the country, not enough to meet the demand[31].

Since the report's publication in 2008, JANGKAR and other NGOs representing injecting drug users have met with senior police and AIDS policymakers to share the report findings and make recommendations for policy reforms. In early 2009, a Supreme Court Memo instructed judges to send drug users to rehabilitation, not prison. While this does not overturn existing sentences of drug users currently in prison, it is still an important step forward[32]. Drug user NGOs are now lobbying actively to persuade judges to begin sentencing drug users to rehab, and are discussing expanded human rights training for district-level officers.

As the newest victims of longstanding weaknesses in Indonesia's police system, Indonesia's drug user NGOs have in some ways become the cutting edge of the human rights movement there. Local harm reduction NGOs in many regions of the country have begun meeting with local police officers to demand treatment access in detention at local facilities, and to raise concerns about the safety of individual detainees. Their advocacy at the local and national level has the potential to both bolster Indonesia's harm reduction movement, and to advance rights protections for everyone detained in the country's criminal justice system.

## Competing interests

SD, AT and RA declare that they have no competing interests.

## Authors' contributions

AT and RA participated in design and coordination of the study and with compiling the research findings. SD prepared the manuscript.
